# The Psychological Impact of Movement Restriction during the COVID-19 Outbreak on Clinical Undergraduates: A Cross-Sectional Study

**DOI:** 10.3390/ijerph17228522

**Published:** 2020-11-17

**Authors:** Aida Kalok, Shalisah Sharip, Abdul Muzhill Abdul Hafizz, Zulkifli Md Zainuddin, Mohamad Nasir Shafiee

**Affiliations:** 1 Department of Obstetrics and Gynaecology, Faculty of Medicine, National University of Malaysia, Universiti Kebangsaan Malaysia Medical Center, Cheras 56000, Malaysia; aidahani.mohdkalok@ppukm.ukm.edu.my (A.K.); muzhill_hafizz@yahoo.com (A.M.A.H.); 2 Department of Psychiatry, Faculty of Medicine, National University of Malaysia, Universiti Kebangsaan Malaysia Medical Center, Cheras 56000, Malaysia; shalisah@ppukm.ukm.edu.my; 3 Department of Surgery, Faculty of Medicine, National University of Malaysia, Universiti Kebangsaan Malaysia Medical Center, Cheras 56000, Malaysia; zuluro@ppukm.ukm.edu.my

**Keywords:** COVID-19, depression, anxiety, stress, students

## Abstract

The COVID-19 pandemic has resulted in a Movement Control Order (MCO) in Malaysia and the subsequent closure of all educational institutions. We aimed to examine the psychological impact of the MCO among clinical undergraduates. A cross-sectional study was conducted using self-reported questionnaires that were distributed online using the Depression, Anxiety and Stress Scale-21 (DASS 21), Short Warwick Edinburgh Mental Well-Being Scale (SWEMWBS), and the newly designed MCO effect questionnaire. Seven hundred seventy-two students completed the survey. The prevalence of psychological distress was 52.8%, with around 60% of respondents reporting disruption to their daily lives. Older (*p* = 0.015) and more senior students (*p* < 0.001) were less likely to be anxious than their younger and junior counterparts, respectively. A greater number of social support (three or more) was linked to a lower score of depression (*p* = 0.005) and stress (*p* = 0.045). Undergraduates who received family support demonstrated lower depression scores (*p* = 0.037) and higher mental wellbeing (*p* = 0.020) compared to those without. Government support was independently associated with a lesser risk of depressive symptoms (Adjusted odds ratio, AOR 0.68; 95% confidence interval, CI 0.47–0.99) and a greater sense of mental wellbeing (AOR 1.54; 95% CI 1.06–2.22). The present finding provides evidence of a high prevalence of psychological distress among clinical undergraduates during the COVID-19 pandemic. Appropriate social support is important in alleviating anxiety and stress and promoting greater mental wellbeing amongst students during the nationwide quarantine.

## 1. Introduction

The Corona Virus Disease 2019 (COVID-19) outbreak has been deemed a global health emergency [1]. To date, there were around 42 million cases diagnosed worldwide, with over one million recorded deaths [2]. Malaysia alone had approximately twenty-six thousand confirmed cases with a mortality rate of 0.86% [3]. Several public health agencies, including the World Health Organization (WHO) and US Centers for Disease Control and Prevention (CDC) have issued advice on preventing further spread of COVID-19, such as social distancing, hand hygiene, and the use of face masks [4,5]. On a broader scale, many countries, including Malaysia, have imposed stringent movement restrictions as infection control measures. The Malaysian Government instigated the Movement Control Order (MCO) on the 18th of March 2020. The MCO, often referred to as a partial lockdown, prohibits mass movements and gatherings at all places nationwide, including religious services. Educational institutions were closed in addition to all government and private premises, except providers of essential services, including food, health, telecommunication, and transportation [6].

For clinical students, a hospital posting puts them at risk of contracting the disease and making the students potential vectors for COVID-19 [7,8]. More immediate concerns among medical undergraduates center on the impact of COVID-19 on their education. Clinical attachments, which involved face-to-face teaching as well as patient exposure, had been halted. The situation can be more complex for those who are due to sit their final exams. 

The emergence of a new pandemic and the unprecedented movement restrictions will lead to anxiety and fear amongst the population. A study on the general population from China found that 28.8% and 16.5% of subjects reported moderate to severe anxiety and depression symptoms, respectively, during the COVID-19 outbreak [9]. Despite several studies on the pandemic’s psychological effect on university and college students [10,11,12,13], the research involving clinical-based undergraduates is still limited [14,15]. Around a fifth of college students in China reported feeling anxious during the COVID-19 outbreak, while university students in Bangladesh demonstrated an alarmingly high anxiety level at 92% [10,12]. A recent study among Malaysian university students revealed that around 29.8% of respondents reported anxiety symptoms that ranged from mild to extremely severe [13]. Li et al. found that 11.1% of health professional students displayed acute stress reactions [15] while a survey amongst medical students in Turkey found that over half of them reported feeling mentally unwell [14].

We aimed to evaluate the movement restriction (MCO) psychological impact during the COVID-19 outbreak among clinical undergraduates in a Malaysian teaching hospital. We hypothesized that the MCO is associated with an increased level of psychological distress among our students and is linked to poorer mental wellbeing. We also hypothesized that the amount of social support received by students is negatively correlated with the stress and anxiety experienced by them.

## 2. Materials and Methods

### 2.1. Study Design and Participants

This was a cross-sectional study involving the Faculty of Medicine students at the National University of Malaysia, Kuala Lumpur. Ethical approval was obtained from the Universiti Kebangsaan Malaysia (UKM) Medical Research and Ethics Committee (Research Code: JEP-2020-035). We conducted an online survey in April 2020 during the fourth and fifth weeks of the MCO. The respondents consisted of university students of different courses, which involved clinical posting (clinical undergraduates), i.e., medicine, nursing, and emergency medical services (paramedics). The sample size calculation was based on the study by Aker et al. [14]. Based on a 5% precision, 95% confidence interval, and 20% dropout, we would need a minimum sample of 460 students. The self-administered questionnaires were distributed to our students via Google Forms, which included a consent section. The study recruitment was conducted through the WhatsApp groups with the help of the student representatives, and the invited participants were asked to complete the online questionnaires over a two-week period. A reminder was sent to the students a week after the initial distribution of the questionnaire.

### 2.2. Instruments

The self-report questionnaires collected socio-demographic data (age, gender, ethnicity), university course, and year of study. The psychological impact of MCO was evaluated through the following measures.

#### 2.2.1. Depression, Anxiety and Stress Scale-21 (DASS 21) and Psychological Distress

The 21-item depression anxiety stress scale (DASS-21) is a self-reporting tool measuring characteristic attitudes and symptoms of depression, anxiety, and stress. DASS-21 is a shortened version of the original 42-item scale (DASS-42) [16]. There are seven items for each emotional state. The items in the depression scale (3, 5, 10, 13, 16, 17, and 21) focused on low mood, low self-esteem, and a poor outlook for the future. The anxiety scale items (2, 4, 7, 9, 15, 19, and 20) concentrated on the fear response and psychological arousal, while the stress subscale items (1, 6, 8, 11, 12, 14, and 18) focused on persistent arousal and tension [17]. The DASS-21 is a reliable, easy-to-use screening instrument and has been well received globally. The Malay version of the questionnaires had been validated and demonstrated to have good psychometric properties for the general Malaysian population [18] and medical students [19]. The Malay DASS-21 had good reliability coefficients (Cronbach’s alpha) for all three subscales; depression (0.863), anxiety (0.850), stress (0.837), and overall (0.90) [20]. Both English and Malay versions of DASS-21 were used in our survey.

The students were asked to rate the extent to which they have experienced various symptoms over the past week, and calculations of the score were conducted based on previous study [21]. The depression scale score was divided into normal (0–9), mild depression (10–12), moderate depression (13–20), severe depression (21–27), and extremely severe depression (28–42). The anxiety scale score was categorized into normal (0–6), mild anxiety (7–9), moderate anxiety (10–14), severe anxiety (15–19), and extremely severe anxiety (20–42). The stress scale scoring was normal (0–10), mild stress (11–18), moderate stress (19–26), severe stress (27–34), and extremely severe stress (35–42).

A respondent who demonstrated symptoms of depression, anxiety, or stress from the calculated score would be considered as experiencing psychological distress.

#### 2.2.2. Short Warwick Edinburgh Mental Well-Being Scale (SWEMWBS)

The Short Warwick–Edinburgh Mental Wellbeing Scale (SWEMWBS) is a shorter version of the 14-items Warwick–Edinburgh Mental Wellbeing Scale (WEMWBS), which was originally developed to monitor wellbeing in the general population, university students [22], and to evaluate policies addressing wellbeing [22,23,24]. There are seven positively worded items, each with five response categories (1, none of the time; 5, all of the time). The score range is 7–35, and higher scores indicate greater mental wellbeing. The English version of the SWEMWBS demonstrated good internal validity with Cronbach’s alpha of 0.89 [25]. The Malay version of the SWEMWBS had been validated, and permission to use it was obtained from the author [26]. We included the English questionnaires alongside the Malay-translated version in our survey.

#### 2.2.3. Perception of the Effect of MCO on Self-Wellbeing

We developed our own survey of the students’ perception of the MCO effect, based on literature and discussion with experts such as psychologists and clinical lecturers. In this survey, three statements were used to assess the students’ perception of MCO’s effect on themselves. The participants were asked to rate each statement using a scale; 1 (strongly disagree) to 10 (strongly agree):

1. MCO has disrupted your daily life.
2. MCO has affected your physical wellbeing.
3. MCO has affected your mental/psychological wellbeing.

The individual statement response score was categorized into strongly agree (1–2), agree (3–4), neutral (5–6), disagree (7–8), and strongly disagree (9–10). The total score for all three responses was calculated, and a cut off level based on the mean score was determined [27,28]. A total score above this level would indicate that the MCO was perceived to have a negative effect on the student. The reliability coefficient (Cronbach’s alpha) of this questionnaire was 0.86.

#### 2.2.4. Source of Social Support

In order to assess the role of social support amongst our students during MCO, we had asked the participants to indicate their perceived source of support. We had listed family, friends, course-mates, university, and government for them to choose from. The students were allowed to choose as many sources as they deemed relevant. The form of support may come in various forms, such as emotional, physical, and financial. We had listed the Malaysian Government as a source of social support because of the measures taken by the authority to help the university students, such as financial aid, free mobile internet allowance, and interstate travel arrangement. The reliability coefficient (Cronbach’s alpha) of this questionnaire was 0.67.

#### 2.2.5. Questionnaire Validation of Social Support and Perception of MCO on Self-Wellbeing

We performed face validation on 15 pre-registration house officers before distributing the questionnaire, which was not included in the final analysis. All of the items underwent exploratory factor analysis to confirm the number of factors, while the reliability of the questionnaire was acquired from the Cronbach alpha value mentioned above.

### 2.3. Statistical Analysis

The Statistical Package for Social Sciences; IBM SPSS Statistics for Windows, version 24 (IBM Corp., Armonk, NY, USA) was used to analyze the study data. Data were presented as mean (standard deviation, SD) or number (percentage) for continuous and categorical variables, respectively. The scores for DASS 21, SWEMBS, and MCO effects were inspected for normality using the Kolmogorov–Smirnov test [29]. Based on the histogram, all data were normally distributed. The internal consistency for the MCO effect measures was assessed using a reliability test, and the Cronbach Alpha for all three items was 0.862. The mean of the SWEWMBS score was used to categorize the scoring into a lower and higher sense of wellbeing. The correlations between the mental wellbeing and depression, anxiety, stress, and MCO effects, were assessed through Pearson’s correlation. The Student’s t-tests and one-way ANOVA were used to determine the differences in mean scores between groups for demographic variables and social support.

A univariate analysis was used to explore the significant associations between the student’s characteristics and depression, anxiety, stress, mental wellbeing, and the MCO effect. Statistically significant variables were screened and included in multivariate logistic regression analyses to produce the adjusted odds ratios (AORs) and the corresponding 95% confidence intervals (CIs). The adjusted odds ratio for individual social support was also determined following adjustment for age and gender. All significant results were based on *p* < 0.05.

## 3. Results

### 3.1. Demographics

We distributed the questionnaires to 993 undergraduates, and 772 of them completed the survey, making the response rate 77.7%. The majority of them were female (71.6%) and below 25 years old (86.0%). Around four-fifths of our respondents were medical students, while the remaining were made up of students from the nursing (13.5%) and emergency medical services course (5.3%). The demographics and university course details are shown in Table 1.

### 3.2. Psychological Distress

Around 52.8% of our students demonstrated psychological distress. The percentages of clinical undergraduates exhibiting symptoms of depression, anxiety, and stress were 36.0%, 44.6%, and 27.6%, respectively. Table 1 depicts the mean scores for DASS-21 for various demographics and social support, while Table 2 demonstrates the prevalence of symptoms according to the different categories. Younger students demonstrated higher level of anxiety (*p* = 0.011) and stress (*p* = 0.015). Malay undergraduates recorded greater stress mean scores than those of Chinese ethnicity. Medical and nursing students showed a higher prevalence of depressive and stress symptoms than emergency medicine students. There were also significant differences observed in anxiety (*p* = 0.002) and stress (*p* = 0.041) levels amongst students of the different years of study.

### 3.3. Mental Wellbeing

The mean and median scores of the SWEMWBS for our cohort were 26.46 and 27.00, respectively. Four hundred sixty-five students (60.2%) scored 26 and above, which indicated high mental wellbeing. The mental wellbeing was negatively correlated with depression, anxiety, and stress (Table 3). Emergency medicine students showed a higher mean wellbeing score than those studying medicine and nursing. There was no significant variation in the wellbeing score based on age, gender, ethnicity, or year of study (Table 1).

### 3.4. MCO Effect on Self-Wellbeing

The response to the 3-items questionnaire on the MCO effect is shown in Table 4. The mean (SD) of the total score was 16.71(7.97) whilst the median score was 16.50. We used the cut-off score of 16 for negative effects, which involved 410 (53.1%) students. Almost 60% of respondents felt that the MCO had disrupted their daily lives. The proportions of students who felt that the MCO had affected their physical and mental wellbeing were 38.6% and 33.8%, respectively. There was no significant correlation between mental wellbeing and the MCO effect (*p* = 0.729) (Table 3).

### 3.5. Social Support

We found that students with a greater amount of social support (3 or more) had a lower score for depression (*p* = 0.005) and stress (*p* = 0.045) (Table 1). These students also demonstrated a higher mental wellbeing score than those with less social support (*p* = 0.001). Analysis into types of support revealed that students who received family support demonstrated a lower score in depression (*p* = 0.037) and higher mental wellbeing (*p* = 0.020) compared to those without. Students who received support from course-mates, university, and the government were also found to have a significantly lesser score in depression.

Government support was significantly associated with lower anxiety (*p* = 0.012) and stress score (*p* = 0.001). Students supported by their course-mates also demonstrated lower stress scores in comparison to those without (*p* = 0.03). Greater mental-wellbeing scores were also observed amongst those who received support from course-mates, university, and the government. Lack of support of family and government was associated with higher MCO effect scores, suggesting a negative influence.

### 3.6. Significant Factors

Table 5 demonstrates the adjusted odds ratios of various factors on depression, anxiety, stress, mental wellbeing, and the MCO effect. Our survey found that older (above the age of 25) (AOR 0.56; 95% CI 0.55–0.89, *p* = 0.015) and more senior students (AOR 0.55; 95% CI 0.41–0.74, *p* < 0.001) were less likely to be anxious. Senior students were 30% less likely to suffer from stress than their junior counterparts (AOR 0.69; 95% CI 0.50–0.96, *p* = 0.027). Participants who received three or more social support types were less likely to demonstrate depressive symptoms (AOR 0.72; 95% CI 0.53–0.97, *p* = 0.028) and 1.4 times likely to demonstrate higher mental wellbeing (AOR 1.41; 95% CI 1.05–1.89, *p* = 0.021). Female students were 44% less likely to experience the MCO’s negative effect than male undergraduates (AOR 0.56; 95% CI 0.40–0.77, *p* < 0.001).

In terms of social support, family support was independently associated with a 65% lesser risk of depressive symptoms (AOR 0.35; 95% CI 0.14–0.84, *p* = 0.019) and greater odds of higher mental-being (AOR 2.82; 95% CI 1.15–6.91, *p* = 0.023), as depicted in Table 6. Interestingly, government support was associated with higher mental wellbeing (AOR 1.54; 95% CI 1.06–2.22, *p* = 0.022) as well as reduced risk of depression (AOR 0.68; 95% CI 0.47–0.99, *p* = 0.043) and stress symptoms (AOR 0.53; 95% CI 0.35–0.80, *p* = 0.003).

## 4. Discussion

The COVID-19 pandemic and the unprecedented movement control order had significant economic and psychosocial repercussions. Our research contributes towards the knowledge gap in the psychological effect of nationwide movement restrictions on clinical undergraduates. Our study found that over half of the students reported symptoms of psychological distress. Younger students (below the age of 25) were more likely to be anxious while senior students displayed lower anxiety and stress level. Around three-fifths of our cohort reported that the MCO had affected their daily lives. Students with a greater amount of social support demonstrated a lower score of depression and stress. Family and government support were independently associated with a lesser risk of depressive symptoms and a greater sense of mental wellbeing.

A study from China reported that the prevalence of depression, anxiety, and stress among the public, during the COVID-19 outbreak was 30.3%, 36.4%, and 32.1%, respectively [9]. The rate of depressive symptoms amongst our cohort was slightly higher at 36.0%. However, this figure was lower than that reported by a community survey at a Spanish university (48.0%). Odriozola-González et al. reported that the prevalence of anxiety and stress amongst university students and the staff was 35.0% and 40%. The Spanish study also found that the Health Sciences students had significantly higher total DASS21 scores than Engineering students [11]. The high prevalence of anxiety (44.6%) amongst our cohort could be explained by the fact that the majority of our students were nursing and medical undergraduates who underwent clinical postings in the hospital.

We found that the students above the age of 25 demonstrated a lower level of anxiety and stress during MCO. The age factor remained significant for anxiety but not stress following multivariable analysis. A previous study conducted amongst Malaysian University students showed that older students had greater anxiety and stress scores than the younger ones [21]. However, the upper age limit for the afore-mentioned study was 24. We also discovered that junior students were more anxious and stressed in comparison to their senior counterparts. Interestingly, although statistically non-significant, the mean score for all three DASS-21 components was higher amongst final year students (year 5) than those in year 3 or 4 of study. Uncertainty about clinical rotations, final exam and graduation, and future job prospects are most likely the contributory factors.

Our study showed that nursing students reported a higher mean score for DASS-21 than the medical and emergency medicine undergraduates. Pooled analysis of studies into health care workers’ mental health during the COVID-19 outbreak revealed that the nurses displayed greater rates of depression and anxiety compared to the doctors. Nursing staff may face a higher risk of exposure to COVID-19 as they spend more time providing direct care to the patients and are responsible for sample collection for viral screening. This occupational hazard may explain the elevated level of anxiety and stress amongst them [29].

The gender gap in the depression prevalence worldwide was well-established and thought to stem mainly from biological sex differences and depends less on culture, ethnicity, and education [30]. A meta-analysis on the prevalence of depression and anxiety amongst healthcare workers during the COVID-19 pandemic found that female healthcare professionals exhibited higher rates of affective symptoms compared to male colleagues [29]. Shamsuddin et al. observed that female university students in Malaysia demonstrated higher stress levels than male undergraduates [21]. Although we did not detect any significant difference between genders amongst our cohort, the female students displayed higher mean scores for all three-subscales of DASS21. Malay students reported significantly higher stress scores than the Chinese, which was consistent with another Malaysian study and most likely contributed by cultural difference [21].

Social support plays an essential role in mitigating the risks of psychological distress amongst university students. Parental and siblings support were significantly associated with the reduced prevalence of emotional disorders amongst medical students [31]. A study from China on health professional students during the COVID-19 outbreak quarantine illustrated that good family functioning was associated with a lesser burden of psychological distress and decreased the risk of acute stress reaction by more than 50% [15]. Our results demonstrated that family support was independently associated with a reduced risk of depression and a greater sense of mental wellbeing.

Our findings showed that government support was significantly linked to higher mental wellbeing with a lower risk of depression and stress. The Malaysian Government had initiated proactive measures to control the COVID-19 pandemic since the beginning of the disease emergence in the country [32]. Specific COVID hospitals were designated to handle positive cases as measures to isolate infective patients while the diagnostic capacity of nationwide laboratories was enhanced to cope with increasing cases. Enforcement of the movement control order (MCO) was the government’s biggest decision to break the chain of COVID-19 within the community. To lessen the economic impact of COVID-19, the government had allocated a huge budget to initiate people-based growth and quality investment. To ensure proper dissemination of information, there was a daily briefing on the MCO and COVID-19 cases by the State Security Council and Ministry of Health, respectively. For university students, the authority had provided a one-off allowance of RM 200 and co-ordinated specific inter-state travel for students to return to their hometown during the MCO. Free daily 1GB internet provision was also helpful for online learning sessions.

The government actions were well received as a recent public survey showed that around 90% of respondents felt that the Malaysian government was handling the health crisis very well and were confident that the country would win its battle against COVID-19 [33]. Government support also yielded positive results amongst undergraduates, as reflected by our study.

We found that higher mental wellbeing was linked with a greater amount of social support. The MCO had inevitably led to social isolation and support from multiple sources, which may include online or virtual communication is important to alleviate emotional distress. An observational study on medical students in Kolkata, India, found a direct correlation between mental wellbeing and social networking scores. Nandi et al. demonstrated that non-stressed individuals had a greater score in the revised Lubben social networking scale (LSNS-R), which assessed the perceived social support from family and friends. The same group also exhibited higher mental wellbeing in comparison to the stressed students [34]. Nandi et al. also found a significant negative correlation between the WEMWBS and General Health Questionnaire (GHQ-28), which subscales include depression, anxiety, somatic symptoms, and social withdrawal. We also found a similar correlation between the SWEMWBS and DASS-21.

We did not find a significant correlation between mental wellbeing and the MCO effect. This may be due to a small number of items in the MCO effect questionnaires or the study duration was not long enough to establish MCO’s negative effect on wellbeing. In a separate analysis, the MCO effect was found to correlate with all the subscales of DASS21, particularly the stress component, which was positively significant (*p* = 0.03). Despite the limited number of items involved, the MCO questionnaire was demonstrating good internal consistency with a good Cronbach Alpha.

### Strengths and Limitations

Our study is the first to assess the psychological impact of COVID-19 and movement restriction on Malaysia’s clinical undergraduates. The large sample size makes our findings clinically significant. The MCO effect questionnaires were the first to evaluate the student perception towards the Malaysian Government’s unprecedented movement control order. This is the first study to assess the mental wellbeing amongst clinical students during the COVID-19 pandemic.

The convenient sampling method through online applications may result in selection bias amongst students with limited internet access. Over two-thirds of our cohort was made up of female students, and this could have contributed to the high prevalence of psychological distress, and current study findings may not be generalized to a cohort with equal gender distribution or postgraduate students. The absence of baseline figures for pre-existing depression and mental wellbeing among our students, which act as controls, also limits our study. Our cross-sectional study conducted approximately one month into the MCO may not detect the true effect of MCO.

Finally, our newly designed questionnaires (social support and MCO effect) are not fully validated, and this should be taken into consideration in the interpretation of our study results. Although both tools can describe the relationship between psychological distress and support as well as the effect of the MCO among clinical undergraduates, further study is needed to determine these questionnaires’ strength, especially in the test-retest reliability.

The high prevalence of psychological distress amongst Malaysian clinical undergraduates during the COVID-19 pandemic highlights the need for an appropriate support system for this cohort of students. The university administration, alongside healthcare professionals, plays an essential role in increasing awareness, conducting regular screening, and providing early treatment or intervention programs to prevent mental health deterioration among students. A longitudinal multicentre study would allow a more comprehensive assessment of the psychological impact of prolonged confinement on students and the effect of timely mental health intervention.

## 5. Conclusions

Our survey demonstrated that the prevalence of psychological distress during movement restrictions amongst clinical students was high. Younger and junior undergraduates were more likely to suffer from anxiety and may benefit from extra support and reassurance. Social support is very important, especially during nationwide quarantine, and greater support was linked with a lesser risk of depression and higher mental wellbeing. Movement restrictions would result in social isolation; hence, effective communication through social media or other forms of virtual networks is essential to provide much needed emotional support, especially from family and friends. Government plays an important role in providing financial and logistic support and disseminating accurate information to the public, which alleviates anxiety and stress within the society and promotes greater mental wellbeing amongst the population.

## Figures and Tables

**Table 1:** Comparison of mean scores based on demographics, university course, and social support.

Factors	*n* (%)	Mean (SD)				
		Depression	Anxiety	Stress	Wellbeing	MCO Effect
**All**	772 (100)	4.06 (4.49)	4.15 (4.29)	5.44 (4.65)	26.46 (5.10)	16.71 (7.97)
***Age***						
**<25**	664 (86.0)	4.24 (4.41)	4.31 (4.28)	5.61 (4.59)	26.36 (5.04)	16.44 (7.81)
**≥25**	107 (13.9)	3.71 (4.97)	3.18 (4.22)	4.43 (4.93)	27.02 (5.43)	18.29 (8.81)
		*p* = 0.255	*p* = 0.011	*p* = 0.015	*p* = 0.218	*p* = 0.043
***Gender***						
**Male**	219 (28.4)	4.02 (4.63)	3.85 (4.53)	5.11 (4.99)	26.92 (5.32)	18.58 (8.03)
**Female**	553 (71.6)	4.22 (4.43)	4.27 (4.19)	5.58 (4.51)	26.28 (5.00)	15.97 (7.83)
		*p* = 0.569	*p* = 0.223	*p* = 0.222	*p* = 0.113	*p* < 0.001
***Ethnicity***						
**a Malay**	470 (60.9)	4.46 (4.62)	4.43 (4.51)	5.81 (4.75)	26.10 (5.07)	16.83 (8.32)
**b Chinese**	134 (17.4)	3.60 (4.13)	3.48 (3.96)	4.62 (4.25)	27.06 (4.80)	15.31 (7.09)
**c Indian**	117 (15.2)	3.91 (4.46)	3.81 (3.84)	5.07 (4.64)	26.81 (5.38)	17.60 (7.65)
**d Other**	51 (6.6)	3.49 (4.12)	4.14 (3.92)	5.08 (4.49)	27.37 (5.27)	17.20 (7.36)
		F (3,768) = 1.884	F (3,768) = 2.025	F (3,768) = 2.769	F (3,768) = 2.130	F (3,768) = 1.960
		*p* = 0.131	*p* = 0.109	*p* = 0.041	*p* = 0.095	*p* = 0.119
				a > b		
***University Course***						
**a Medicine**	627 (81.2)	4.19 (4.54)	4.04 (4.25)	5.41 (4.64)	26.32 (5.03)	16.82 (7.83)
**b Nursing**	104 (13.5)	4.73 (4.39)	5.43 (4.58)	6.49 (4.78)	26.15 (5.27)	15.59 (8.31)
**c Emergency Medical Services**	41 (5.3)	2.34 (3.38)	2.66 (3.32)	3.37 (3.69)	29.41 (4.94)	17.78 (9.03)
		F (2,769)= 4.255	F (2,769) = 7.465	F (2,796) = 6.853	F (2,769) = 7.440	F (2,769) = 1.466
		*p* = 0.015	*p* = 0.001	*p* = 0.001	*p* = 0.001	*p* = 0.232
		a > c; b > c	b > a; b > c	a > c; b > c	c > a; c > b	
***Year of Study***						
**a 1**	171 (22.2)	4.44 (4.46)	4.95 (4.04)	5.99 (4.35)	26.47 (5.29)	16.99 (8.02)
**b 2**	163 (21.1)	4.47 (4.25)	4.76 (4.32)	5.99 (4.57)	26.72 (4.73)	16.36 (8.15)
**c 3**	142 (18.4)	4.02 (4.48)	3.37 (3.90)	4.73 (4.56)	26.73 (5.66)	16.25 (8.14)
**d 4**	155 (20.1)	3.45 (4.01)	3.54 (4.21)	4.92 (4.63)	26.66 (4.64)	16.10 (7.28)
**e 5**	141 (18.3)	4.40 (5.22)	3.95 (4.78)	5.45 (5.07)	25.66 (5.14)	17.89 (8.23)
		F (4,767) = 1.470	F (4,767) = 4.417	F (4,767) = 2.511	F (4,767) = 1.134	F (4,767) = 1.251
		*p* = 0.209	*p* = 0.002	*p* = 0.041	*p* = 0.339	*p* = 0.288
			a > c; a > d; b > c			
***No of support***						
**1–2**	409 (53.0)	4.59 (4.70)	4.43 (4.48)	5.76 (4.84)	25.90 (5.37)	16.62 (7.95)
**3 or more**	363 (47.0)	3.68 (4.19)	3.85 (4.04)	5.09 (4.40)	27.09 (4.70)	16.80 (8.00)
		*p* = 0.005	*p* = 0.059	*p* = 0.045	*p* = 0.001	*p* = 0.760
**Family support**						
**Y**	750 (97.2)	4.07 (4.38)	4.12 (4.24)	5.41 (4.61)	26.54 (5.03)	16.59 (7.93)
**N**	22 (2.8)	7.27 (6.70)	5.41 (5.68)	6.77 (5.92)	23.59 (6.54)	20.59 (8.71)
		*p* = 0.037	*p* = 0.301	*p* = 0.174	*p* = 0.020	*p* = 0.007
**Friends support**						
**Y**	465 (60.2)	3.95 (4.29)	4.02 (4.05)	5.33 (4.45)	26.65 (5.06)	16.91 (8.04)
**N**	307 (39.8)	4.48 (4.77)	4.35 (4.62)	5.62 (4.94)	26.17 (5.15)	16.40 (7.87)
		*p* = 0.110	*p* = 0.295	*p* = 0.397	*p* = 0.199	*p* = 0.388
**Course-mates**						
**Y**	335 (43.4)	3.74 (4.30)	3.82 (4.12)	5.03 (4.42)	27.12 (4.77)	17.04 (8.06)
**N**	437 (56.6)	4.49 (4.61)	4.41 (4.41)	5.76 (4.80)	25.95 (5.29)	16.45 (7.90)
		*p* = 0.020	*p* = 0.060	*p* = 0.030	*p* = 0.001	*p* = 0.314
**University**						
**Y**	216 (28.0)	3.41 (3.86)	3.72 (3.88)	5.02 (4.32)	27.35 (4.77)	16.90 (8.10)
**N**	556 (72.0)	4.46 (4.68)	4.32 (4.43)	5.61 (4.76)	26.12 (5.18)	16.63 (7.93)
		*p* = 0.002	*p* = 0.063	*p* = 0.113	*p* = 0.003	*p* = 0.677
**Government**						
**Y**	311 (40.3)	3.40 (3.91)	3.69 (4.05)	4.79 (4.34)	27.29 (4.71)	16.00 (8.08)
**N**	461 (59.7)	4.68 (4.78)	4.47 (4.42)	5.88 (4.80)	25.90 (5.27)	17.18 (7.87)
		*p* < 0.001	*p* = 0.012	*p* = 0.001	*p* < 0.001	*p* = 0.042

**Table 2:** Depression, anxiety, and stress symptoms prevalence according to categories.

DASS-21 Category	Depression *n* (%)	Anxiety *n* (%)	Stress *n* (%)
Normal	494 (64.0)	428 (55.4)	559 (72.4)
Mild	84 (10.9)	59 (7.6)	86 (11.1)
Moderate	111 (14.4)	148 (19.2)	52 (6.7)
Severe	45 (5.8)	48 (6.2)	53 (6.9)
Extremely severe	38 (4.9)	89 (11.5)	22 (2.8)

**Table 3:** Correlations between mental wellbeing and depression, anxiety, stress, and MCO effect.

	Well-Being, *r*	*p* Value
**Depression**	−0.482	<0.001
**Stress**	−0.328	0.001
**Anxiety**	−0.436	<0.001
**MCO Effect**	−0.133	0.729

*r* correlation coefficient.

**Table 4:** Perception of the MCO Effect on self.

	Statement	Response, *n* (%)				
		Strongly Disagree (1–2)	Disagree (3–4)	Neutral (5–6)	Agree (7–8)	Strongly Agree (9–10)
**1**	MCO has disrupted your daily life	99 (12.8)	109 (14.1)	106 (13.7)	200 (25.9)	258 (33.4)
**2**	MCO has affected your physical well-being	199 (25.8)	136 (17.6)	139 (18.0)	145 (18.8)	153 (19.8)
**3**	MCO has affected your mental/psychological well-being	243 (31.5)	137 (17.7)	131 (17.0)	136 (17.6)	125 (16.2)

**Table 5:** Associations between students’ characteristics and social support and depression, anxiety, stress, high wellbeing, and the MCO effect.

Factors	Depression			Anxiety			Stress			Higher Wellbeing			Negative MCO Effect		
	*AOR*	95% CI	*p* Value	*AOR*	95% CI	*p* Value	*AOR*	95% CI	*p* Value	*AOR*	95% CI	*p* Value	*AOR*	95% CI	*p* Value
**Age >25**	0.95	0.60–1.50	0.832	0.56 *	0.35–0.89	0.015	0.84	0.51–1.40	0.507	1.09	0.71–1.69	0.690	1.14	0.74–1.76	0.550
**Female gender**	1.03	0.74–1.44	0.856	1.20	0.86–1.66	0.283	0.98	0.69–1.40	0.915	0.93	0.67–1.28	0.648	0.56 **	0.40–0.77	<0.001
**Senior students (year 4–5)**	0.78	0.57–1.06	0.113	0.55 **	0.41–0.74	<0.001	0.69 *	0.50–0.96	0.027	0.75	0.55–1.01	0.059	1.00	0.74–1.34	0.973
**More support (≥3)**	0.72 *	0.53–0.97	0.028	0.88	0.66–1.18	0.383	0.83	0.60–1.14	0.254	1.41 *	1.05–1.89	0.021	0.98	0.74–1.30	0.883

AOR, adjusted odds ratio; CI, confidence interval; adjusted for age, gender, perceived support and year of study; * *p* < 0.05 ** *p* < 0.001.

**Table 6:** Associations between types of support and depression, anxiety, stress, high mental wellbeing, and MCO’s negative effect.

Support	Depression			Anxiety			Stress			Higher Wellbeing			Negative MCO Effect		
	*AOR*	95% CI	*p* Value	*AOR*	95% CI	*p* Value	*AOR*	95% CI	*p* Value	*AOR*	95% CI	*p* Value	*AOR*	95% CI	*p* Value
Family	0.35 *	0.14–0.84	0.019	0.43	0.17–1.04	0.062	0.76	0.30–1.94	0.572	2.82 *	1.15–6.91	0.023	0.62	0.25–1.57	0.313
Friends	0.89	0.61–1.31	0.561	1.19	0.82–1.73	0.355	1.04	0.70–1.56	0.839	1.01	0.69–1.46	0.979	0.97	0.67–1.41	0.880
Course-mates	0.99	0.66–1.50	0.976	0.77	0.51–1.14	0.191	0.88	0.57–1.37	0.573	1.30	0.87–1.94	0.200	1.06	0.71–1.57	0.791
University	0.88	0.56–1.37	0.572	1.15	0.75–1.77	0.517	1.25	0.77–2.03	0.372	0.88	0.57–1.37	0.578	1.23	0.80–1.88	0.341
Government	0.68 *	0.47–0.99	0.043	0.77	0.54–1.11	0.158	0.53 *	0.35–0.80	0.003	1.54 *	1.06–2.22	0.022	0.70	0.49–1.01	0.054

AOR, adjusted odds ratio; CI, confidence interval; adjusted for age and gender; * *p* < 0.05.
